# 

**Published:** 1903-05

**Authors:** 


Vol. XVIII. MAY, 1903. No. 11.

STATE MEDICAL ASSOCIATION NUMBER.

### TEXAS Medical Journal.

LIBRARY

Subscription, $1.00 a Year, in Advance.

Single Copies, 10 Cents.

PUBLISHED AT AUSTIN, TEXAS, BY
F. E. DANIEL, M. D.,
Proprietor.

Eastern Representative: John Guy Monihan, St. Paul Building, 220 Broadway, New York City.

Entered at the Postoffice at Austin, Texas, as Second-class Mail Matter.

